# Postoperative serum mir-28-5p level has predictive value for the prognosis after endovascular abdominal aortic aneurysm repair

**DOI:** 10.1186/s13019-024-02758-z

**Published:** 2024-04-25

**Authors:** Senyan Wu, Guobing Cheng, Wei Lu, Youyao Xu

**Affiliations:** Department of Vascular Surgery, The Quzhou Affiliated Hospital of Wenzhou Medical University, Quzhou People’s Hospital, 100 Minjiang Avenue, Kecheng District, Quzhou, Zhejiang 324000 China

**Keywords:** Endovascular abdominal aortic aneurysm repair, MicroRNA-28-5p, Abdominal aortic aneurysm diameter, CD3^+^, CD4^+^, CD8^+^, TC, TG

## Abstract

**Background:**

We explored the clinical significance of miR-28-5p pre- and post-endovascular abdominal aortic aneurysm repair (EVAR) in abdominal aortic aneurysm (AAA) patients.

**Methods:**

Subjects included AAA patients receiving EVAR and non-AAA people without statistical differences from AAA patient in comorbidities/Framingham risk score. Fasting elbow venous blood (4 mL) was collected in the morning of the day of EVAR surgery and in the morning of 3 months post-EVAR. Pre-/post-EVAR serum miR-28-5p expression, AAA maximum diameter alterations, CD^3+^/CD^4+^/CD^8+^/TC/TG pre-/post-EVAR, and the correlations between miR-28-5p and AAA maximum diameter were investigated. Prediction of miR-28-5p on post-EVAR mortality, prognosis, and independent factors of post-EVAR death were analyzed using receiver operating characteristic curve (ROC)/Kaplan-Meier curve/univariable and multivariable Cox regression. According to the cut-off value of ROC curve for postoperative miR-28-5p was the cut-off value, and the patients were classified into the miR-28-5p high- and low-expression groups. The survival or death of both groups were compared after 48-month follow-up.

**Results:**

Serum miR-28-5p levels in AAA patients dropped post-EVAR. AAA patients showed notable differences in CD^3+^/CD^4+^/CD^8+^/TC/TG levels pre-/post-EVAR. The miR-28-5p low-expression group exhibited higher CD^3+^/CD^4+^ and lower CD^8+^/TC/TG levels. We observed a positive correlation between post-EVAR miR-28-5p and AAA maximum diameter and between the pre-/post-EVAR miR-28-5p fold change and the AAA maximum diameter change. Postoperative miR-28-5p demonstrated good predictive value for postoperative death. Hypertension, Framingham risk score, TC, TG, and miR-28-5p were independent influencing factors of post-EVAR death.

**Conclusion:**

EVAR decreased serum miR-28-5p expression in AAA patients. Post-operative miR-28-5p level and pre-/post-operative fold change level are positively-correlated with AAA diameter.

**Supplementary Information:**

The online version contains supplementary material available at 10.1186/s13019-024-02758-z.

## Introduction

Abdominal aortic aneurysm (AAA) is characterized by the dilatation and weakening of the abdominal aorta, typically affecting the infrarenal segment [1]. The prevalence of AAA in males aged over 65 is estimated to be 4–8%, while in females aged above 65, the prevalence is 1–2% [2]. It is influenced by a combination of genetic and environmental factors and is associated with significant cardiovascular mortality and morbidity [3]. Of note, ruptured AAA is a critical cardiovascular emergency with a 30-day mortality as high as 70% [4]. Nevertheless, most patients are asymptomatic and their symptoms are often found by accident because they have inadvertently touched an abdominal pulsating mass or by a healthy physical examination. Currently, AAA management options include monitoring or surgical repair [5]. Endovascular abdominal aortic aneurysm repair (EVAR) has emerged as a minimally invasive alternative to open surgery for AAA repair [3]. EVAR has led to a decline in open surgical procedures and has been shown to have lower operative mortality in clinical trials [6, 7]. Therefore, it is of vital importance to investigate the underlying mechanisms of AAA, and evaluating the effectiveness of EVAR is crucial for ameliorating and treating AAA.

microRNAs (miRNAs) are small non-coding RNA molecules, typically 21–23 nucleotides in length, that regulate gene expression by binding to specific mRNA targets, leading to translational inhibition or target degradation [8]. Emerging evidence suggests that miRNAs can modulate the activity of vital cellular components involved in AAA formation, including endothelial cells, fibroblasts, and smooth muscle cells [9]. For instance, miR-424/322 has been shown to protect against AAA formation by regulating the Smad2/3/runt-related transcription factor 2 axis [10]. In contrast, miR-144-3p is highly expressed in AAA tissues and facilitates AAA progression in animal models [11]. More than that, miR-126 has been found to improve cell survival, suppress inflammatory cytokines, and inhibit AAA development in mice [12]. Moreover, miR-28-3p has shown promise as a potential biomarker for predicting the development of AAA in individuals with sub-aneurysmal aortic dilation [13]. It was also documented that CircCBFB-miR-28-5p axis stimulates AAA via GRIA4 and LYPD3 [14]. miR-28-5p has been implicated in various cardiovascular diseases, indicating its potential involvement in AAA [15, 16]. However, there is limited research exploring the expression and clinical significance of serum miR-28-5p in AAA and its pre- and post-EVAR potential changes. As a consequence, the purpose of this study was to investigate the expression and clinical relevance of pre- and post-EVAR serum miR-28-5p.

## Materials and methods

### Study subjects

This study included a total of 120 AAA patients [81 males and 39 females, average age of 68.85 ± 8.75 years, average Body Mass Index (BMI) 23.10 ± 2.00] who underwent EVAR at The Quzhou Affiliated Hospital of Wenzhou Medical University, Quzhou People’s Hospital between January 2018 and December 2019. Among them, there were 55 cases with smoking history and 65 cases with drinking history, including 20 cases of hyperlipidemia, 32 cases of diabetes, 24 cases of chronic obstructive pulmonary disease, 41 cases of hypertension, and 26 cases of coronary heart disease, and the Framingham risk score was 21.00 (16.00, 24.00). Besides, 100 non-AAA people showing no statistical difference in comorbidities and Framingham risk score compared to AAA patients during the same period were selected as the control group (65 males and 35 females, average age of 68.07 ± 7.28 years, average BMI of 22.95 ± 2.24). Wherein 42 cases had a history of smoking and 50 cases had a history of drinking, complicated by 14 cases of hyperlipidemia, 25 cases of diabetes, 20 cases of chronic obstructive pulmonary disease, 33 cases of hypertension, and 21 cases of coronary heart disease, with the Framingham risk score of 21.00 (17.00, 24.00). There were no statistical differences in age, sex, BMI, smoking history, drinking history, comorbidities, or Framingham risk score between the two groups (*P* > 0.05) (Supplementary Table S1).

The inclusion criteria were as follows: (1) definitive diagnosis of AAA confirmed by abdominal aortic computed tomography angiography (CTA); (2) tumor body diameter > 5 cm and with an unruptured tumor body; (3) age ≤ 85 years old; (4) no known allergy to the contrast agent and absence of severe cardiac, liver, or renal dysfunction.

The exclusion criteria were as below: (1) allergy to contrast media and the presence of severe cardiac, liver, renal dysfunction, and respiratory failure; (2) presence of a ruptured abdominal aortic aneurysm with extremely unstable vital signs; (3) severely narrow or tortuous femoral artery that could not facilitate the passage of the stentgrafts; (4) inability to tolerate EVAR; (5) with ruptured aneurysms during hospitalization; (6) EVAR re-interveners; (7) transferred to other departments for surgical treatment during hospitalization; (8) uderwent aortic surgery within one month prior to surgery; (9) taken immunosuppressive drugs within one month prior to surgery; 10) combined with hematological diseases or malignant tumor progression.

### Sample and data collection

Fasting elbow vein blood samples of 4 mL were collected from the control group on the day of the physical examination. As well, 4 mL of fasting elbow vein blood was gathered from all patients with AAA early in the morning on the day of EVAR surgery and early in the morning 3 months post-operation. The collected blood samples were then centrifuged at 2000 r/min for 20 min, and the upper serum was carefully collected and stored in a sterile eppendorf tube at -80 °C for freezing. Demographic information including age, sex, BMI, drinking history, smoking history, complications (hyperlipidemia, diabetes, chronic obstructive pulmonary disease, hypertension, and coronary heart disease), Framingham risk score, and relevant pathological indicators were recorded for each participant. Image scanning was implemented using a Lightspeed Ultra CT scanner (General Electric Company, Schenectady, NY, USA). The data were processed using GE workstation adw 4.4 to acquire the maximum diameter of AAA. The levels of serum T cell subsets (CD3^+^, CD4^+^, and CD8+) were measured using an automatic flow cytometer (Thermo Fisher Scientific, Shanghai, China), while blood lipid levels, including total cholesterol (TC) and triglyceride (TG), were assessed using an automatic biochemical analyzer (Hitachi 917, Roche Diagnostics GmBH, Mannheim, Germany).

### Reverse transcription quantitative polymerase chain reaction (RT-qPCR)

Total RNA content extraction from serum was performed using the TRIzol reagent (Invitrogen, Carlsbad, CA, USA). The extracted RNA content was reverse transcribed into cDNA using the RT kit (Takara, Dalian, Liaoning, China) in strict accordance with the provided instructions. RT-qPCR was performed using SYBR Green real-time PCR Master Mix (Takara) and miScript SYBR Green PCR Kit (Qiagen, Cologne, Germany) on the ABI 7500 fast real-time PCR system (ABI, Foster City, CA, USA). The PCR reaction conditions were as follows: 95 °C 30 s, 64 °C 25 s, 72 °C 30 s, for 35 cycles. The expression of miR-28-5p was determined using the 2^−ΔΔCt^ method after normalization to the internal reference U6. The primer sequences are shown in Table 1.

**Table 1:** RT-qPCR primer sequences

Gene	Forward5’-3’	Reverse5’-3’
miR-28-5p	AAGGAGCUCACAGUCUAUUGAG	CTCGTTCGGCAGCACA
U6	CTCGCTTCGGCAGCACA	AACGCTTCACGAATTTGCGT

### Postoperative follow-up

Regular telephonic follow-ups were conducted every 6 months by the vascular surgeon or designated nurse to assess patients post-operative recovery and survival. The post-operative follow-up duration extended to 48 months, ensuring post-operative comprehensive information on long-term patient survival or death.

### Statistical analysis

SPSS 21.0 (IBM Corp., Armonk, NY, USA), GraphPad Prism 8.01 (GraphPad Software Inc., San Diego, CA, USA), and Medcalc (MedCalc Software Ltd., Belgium) were applied for statistical analysis and mapping. Counting data were expressed in terms of cases or percentages and compared between two groups using Chi-square test. The Shapiro-Wilk test was employed for assessing the normal distribution of continuous variables. Measurement data of normal distribution were presented as mean ± standard deviation and compared using the *t* test between 2 groups. Measurement data of non-normal distribution were presented by the median (minimum, maximum) and compared using the Mann-Whitney U test between two groups. Pearson method was utilized for analyzing the correlations between post-EVAR serum miR-28-5p expression and AAA diameter and the correlation between serum miR-28-5p change. The predictive value of serum miR-28-5p fold change level and AAA maximum diameter change level on post-EVAR death was assessed using the receiver operating characteristic (ROC) curve, and the prognosis was evaluated using the Kaplan-Meier curve. Univariable and multivariable Cox regression analyses were conducted to identify independent influencing factors of postoperative death.

### Ethics approval

The experiments were authorized by the academic ethics committee of The Quzhou Affiliated Hospital of Wenzhou Medical University, Quzhou People’s Hospital(Approval No. 2021-022). All procedures were strictly implemented according to Declaration of Helsinki. All subjects involved were fully informed of the study objective and signed the informed consent before sampling.

## Results

### Endovascular repair diminished serum mir-28-5p expression in AAA patients

**Fig. 1:**
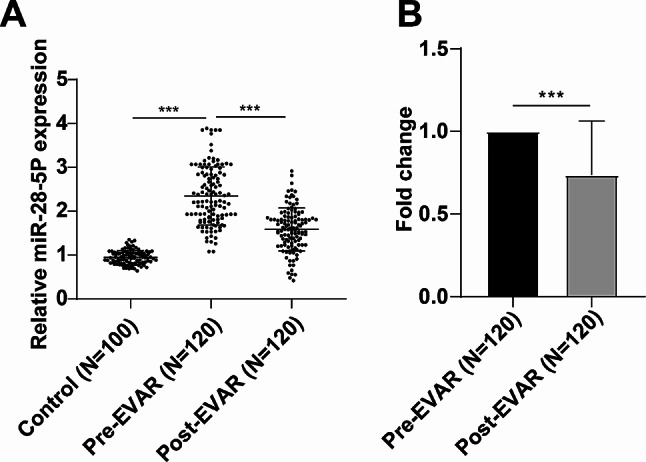
EVAR reduced serum miR-28-5p expression in AAA patients. A: RT-qPCR to examine the pre- and post-EVAR serum miR-28-5p expression levels of the control group and AAA patients; B: Comparison of the fold changes of post-EVAR miR-28-5p levels in AAA patients compared with preoperative miR-28-5p levels. The measurement data of non normal distribution were expressed by the median (minimum, maximum), and compared between two groups using Mann-Whitney U test. *** *P* < 0.001

Prior research has consistently reported high expression levels of miR-28-5p in AAA [14]. RT-qPCR analysis showed that AAA patients exhibited higher serum miR-28-5p fold change levels than the control group, while there was a significant decrease in serum miR-28-5p expression in AAA patients post-operation compared to pre-operation (*P* < 0.001), and the post-EVAR fold change level of miR-28-5p in AAA patients < 1 (Fig. 1A-B). Briefly, these findings indicate that EVAR resulted in a reduction of miR-28-5p expression in the serum of AAA patients.

### The relationship between pre- and post-EVAR serum mir-28-5p expression and clinicopathological parameters

**Fig. 2:**
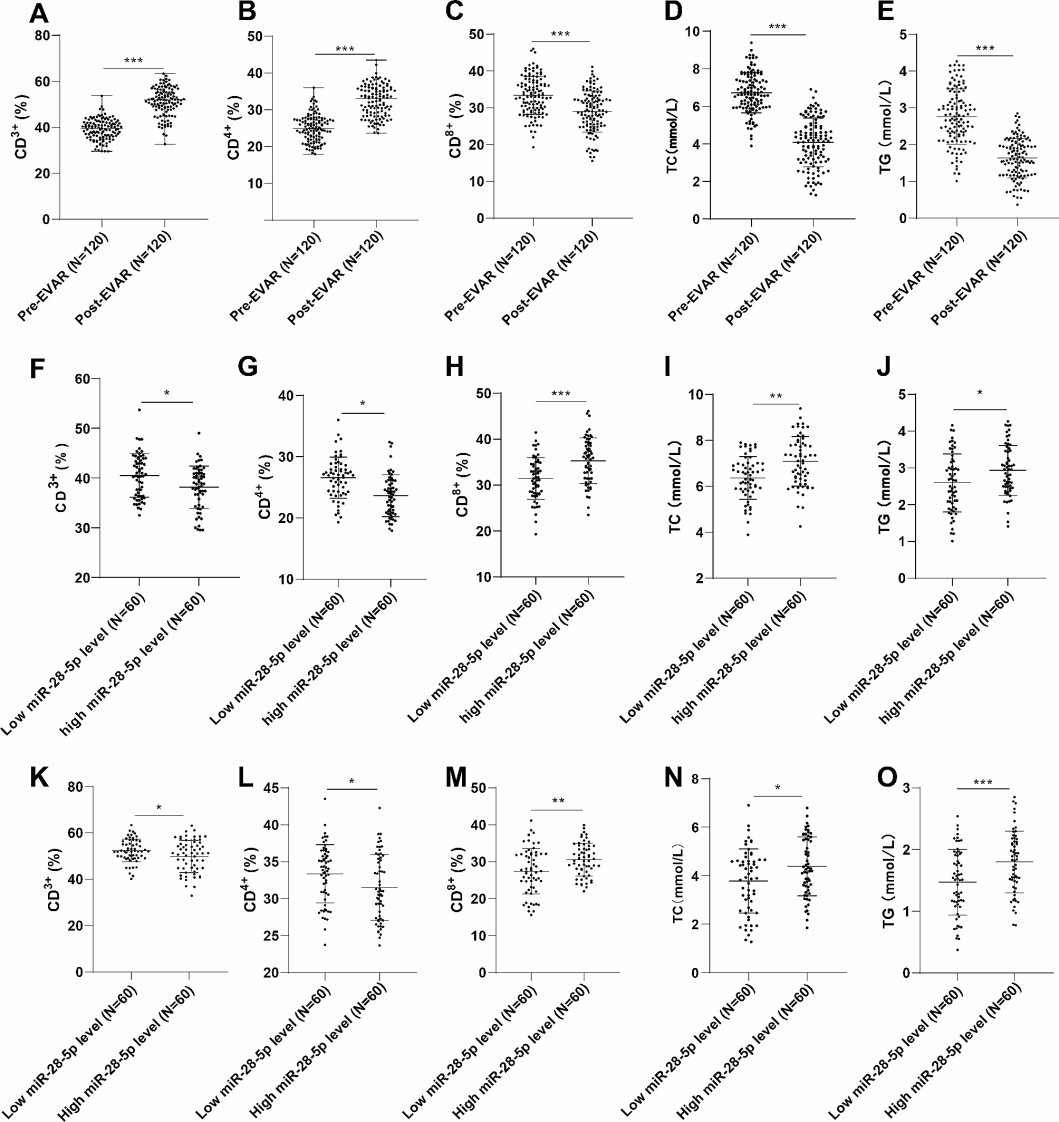
The relationship between post-EVAR serum miR-28-5p levels and clinical pathological indicators. A. B, C, F, G, H, K, L, M: Determinations of serum T cell subsets (CD3^+^, CD4^+^, CD8^+^) levels using fully automated flow cytometry; D, E, I, J, N, O: Assessments of TC and TG levels using fully automatic biochemical analyzer. Note: Non-normal distribution data were represented by median (minimum, maximum), and panels A and B were tested by Mann-Whitney U test. Data conforming to normal distribution were expressed as mean ± standard deviation. Data between two groups in panels C, D, E, F, G, H, I, J, K, L, M, and O were tested by independent sample **t** test. * *P* < 0.05, ** *P* ＜ 0.01, *** *P* < 0.001

To investigate the relationship between pre- and post-EVAR serum miR-28-5p expression and T cell subsets (CD3^+^, CD4^+^, CD8^+^) as well as lipid components (TC, TG), the CD3^+^, CD4^+^, CD8^+^, TC, and TG of AAA patients pre- and post-EVAR were statistically analyzed. As shown in Fig. 2A and E, significant differences were identified in CD3^+^, CD4^+^, CD8^+^, TC, and TG levels between AAA patients pre- and post-EVAR (all *P* < 0.001). Subsequently, we categorized AAA patients into the miR-28-5p high-expression group and the miR-28-5p low-expression group based on the pre-EVAR median serum miR-28-5p value of 2.23 as the cutoff value. It was discovered that the miR-28-5p low-expression group had higher levels of CD3 + and CD4 + and lower levels of CD8+, TC, and TG compared with the miR-28-5p high-expression group (all *P* < 0.05) (Fig. 2F and J). Similarly, we divided the AAA patients into two groups based on the median value (1.60) of post-EVAR serum miR-28-5p: the high miR-28-5p expression group and the low miR-28-5p expression group. In comparison to the high miR-28-5p expression group, the low miR-28-5p expression group exhibited elevated CD3^+^ and CD4^+^ levels, as well as suppressed CD8^+^, TC, and TG levels (all *P* < 0.05) (Fig. 2K and O).

### The correlation between post-EVAR serum miR-28-5p level, pre- and post-operative Fold changes, and tumor diameter

**Fig. 3:**
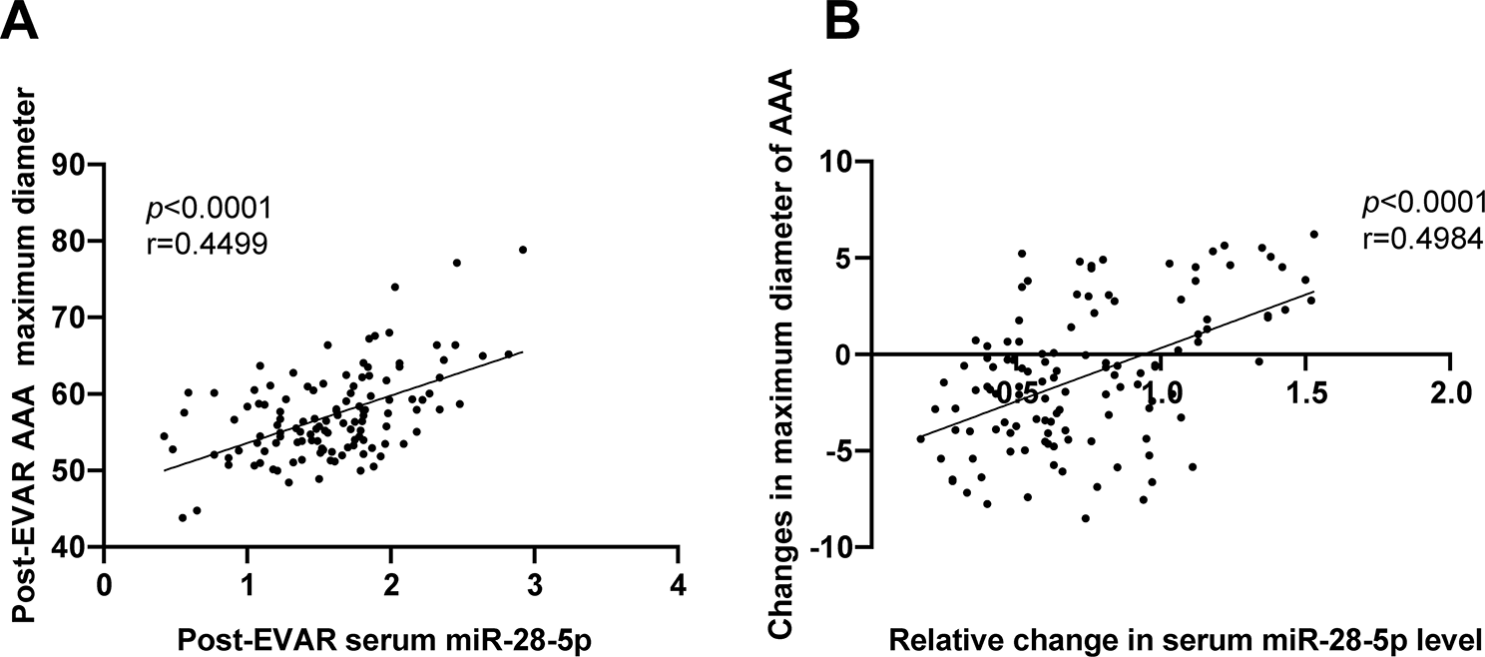
The correlation between post-EVAR serum miR-28-5p level, preoperative and postoperative fold level changes, and tumor diameter. A: Post-EVAR serum miR-28-5p expression and its correlation with AAA diameter; B: Pre- and post-EVAR serum miR-28-5p change level and its correlation with AAA diameter fold change level

Furthermore, we conducted Pearson correlation analysis to examine the relationship between post-EVAR serum miR-28-5p expression and AAA diameter and the correlation between pre- and post-EVAR serum miR-28-5p fold change level and AAA diameter. The results revealed a significant positive correlation between post-EVAR serum miR-28-5p and AAA diameter (*P* < 0.0001, *r* = 0.4499) (Fig. 3A). Moreover, the fold change in pre- and post-EVAR serum miR-28-5p level exhibited a positive correlation with the change in AAA diameter (*P* < 0.0001, *r* = 0.4984) (Fig. 3B).

### The prognostic value of serum mir-28-5p level in EVAR

**Fig. 4:**
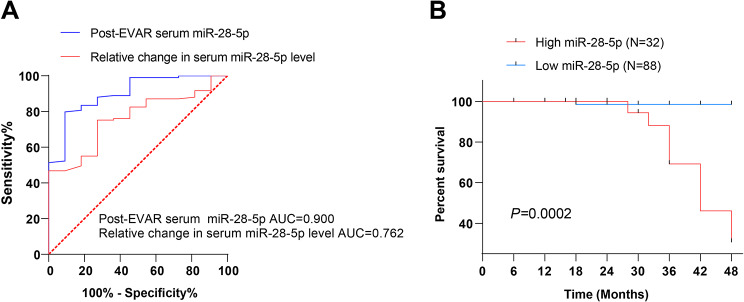
The prognostic value of serum miR-28-5p levels in EVAR treatment. A: ROC curve analyses of postoperative serum miR-28-5p, preoperative and postoperative miR-28-5p fold change levels predicting post-EVAR mortality; B: Prognostic analyses using Kaplan-Meier curve

To determine the prognostic value of serum miR-28-5p level in EVAR, we plotted the ROC curve using the post-surgery serum miR-28-5p and fold change in miR-28-5p level in AAA patients. The area under the ROC curve (AUC) of postoperative miR-28-5p level predicting post-EVAR death was 0.900, with a sensitivity of 79.82% and a specificity of 90.91%, and the cutoff value was 1.845. The AUC of the fold change level of post-EVAR serum miR-28-5p predicting death was 0.762, with 75.23% sensitivity, 72.73% specificity, and a 0.8900 cutoff value (Fig. 4A). The aforesaid findings elucidated that both postoperative serum miR-28-5p and AAA diameter had good predictive value for post-EVAR mortality. In addition, as per the cut-off value of 1.845 in the ROC analysis of postoperative serum miR-28-5p, patients were categorized into high and low expression groups, with the survival or death after 48 months of follow-up of the two groups compared. The prognostic analysis was conducted using the Kaplan-Meier curve. Patients with high postoperative serum miR-28-5p expression were at an even greater likelihood of dying (Fig. 4B).

### Postoperative serum mir-28-5p level was an independent influencing factor for post EVAR death

The univariable and multivariable Cox regression analyses were performed with the efficacy of EVAR as the dependent variable (survival = 1, death = 0) and the general data of AAA patients, the clinicopathologic indexes (CD3^+^, CD4^+^, CD8^+^, TC, TG), and postoperative serum miR-28-5p as independent variables. The univariable analysis showed that BMI, hyperlipidemia, diabetes, chronic obstructive pulmonary disease, hypertension, coronary heart disease, Framingham risk score, CD^3+^, CD^4+^, CD^8+^, TC, TG, and miR-28-5p were significantly associated with post-EVAR death in AAA patients (all *P* < 0.05). The results of multivariable analysis revealed that hypertension, Framingham risk score, TC, TG, and miR-28-5p were identified as independent influencing factors for post-EVAR (all *P* < 0.05, Table 2).

**Table 2:** Univariable and multivariable Cox regression analyses of death after EVAR

Variable	Univariable		Multivariable	
	HR (95% CI)	*P*	HR (95% CI)	*P*
Sex	0.928 (0.199–0.320)	0.924	/	/
Age (years)	1.011 (0.945–1.081)	0.760	/	/
BMI (kg/m^2^)	1.571 (1.156–2.134)	0.004	0.990 (0.500-1.958)	0.977
Smoking history	2.248 (0.592–8.532)	0.234	/	/
Drinking history	2.886 (0.623–13.373)	0.175	/	/
Hyperlipidemia	2.888 (0.088–0.945)	0.040	0.685 (0.079–5.933)	0.731
Diabetes	0.103 (0.022–0.477)	0.004	0.151 (0.007–3.157)	0.223
Chronic obstructive pulmonary disease	0.225 (0.065–0.772)	0.018	0.193 (0.014–2.735)	0.224
Hyperemia	0.240 (0.064–0.896)	0.034	0.009 (0.000-0.572)	0.026
Coronary heart disease	0.075 (0.016–0.348)	0.001	0.085 (0.003–2.542)	0.155
Framingham risk score	2.085 (1.128–3.857)	0.019	0.195 (0.039–0.981)	0.047
CD3^+^ (%)	0.849 (0.777–0.927)	0.000	0.744 (0.528–1.047)	0.089
CD4^+^ (%)	0.752 (0.652–0.911)	0.004	0.562 (0.312–1.014)	0.055
CD8^+^ (%)	1.161 (1.011–1.334)	0.035	0.687 (0.380–1.241)	0.213
TC (× 10^− 1^ mmol/L)	1.147 (1.038–1.268)	0.007	1.371 (1.049–1.793)	0.021
TG (× 10^− 1^ mmol/L)	1.124 (1.072–1.376)	0.002	1.445 (1.022–2.042)	0.037
miR-28-5p (× 10^− 1^)	1.232 (1.086–1.398)	0.001	0.577 (0.352–0.946)	0.029

## Discussion

miRNAs are fairly stable in storage and can be identified in tissues or blood circulation, making them a promising biomarker for AAA [17, 18]. The adjustment of miRNAs and their corresponding target genes is a novel therapeutic approach influencing AAA progression [19]. Consistent with our results, an elevation in miR-28-5p expression has been observed in AAA patients [14]. However, the impact of post-EVAR miR-28-5p in AAA patients has not been previously explored. This study illustrated that EVAR leads to lessened serum miR-28-5p expression in AAA patients, shedding light on the potential role of miR-28-5p in AAA treatment.

T cell subsets, specifically CD3^+^, CD4^+^, and CD8^+^ T lymphocytes, play vital roles in immune regulation and immunoresponse modulation, and their dynamic balance is essential for maintaining homeostasis and influencing the recovery process following surgery [20]. Genetic studies have implicated lipid dysfunction as a significant risk factor for aneurysms, highlighting that the potential lipid-repressing drugs may be a promising therapy for preventing aneurysms [21]. Our results revealed significant differences in the levels of CD3^+^, CD4^+^, CD8^+^, TC, and TG between AAA patients and the control group, as well as the pre- and post-EVAR AAA patients. Moreover, elevated TG and TC levels have been identified in the peripheral blood of AAA patients with aneurysms > 5 cm relative to those with small aneurysms (3–5 cm), suggesting a close association between blood lipid level and AAA development [22]. Notably, our study made a novel observation by demonstrating that AAA patients with lowly-expressed miR-28-5p had elevated levels of CD3^+^ and CD4^+^ T cells and suppressed CD8^+^, TC, and TG levels.

Moreover, there have been reports of negative correlations between human plasma miR-195 levels and AAA diameter [23], along with increased serum miR-29c-3p levels in AAA patients correlating with aneurysm diameter [24]. Subsequent findings revealed a positive correlation between postoperative miR-28-5p levels and AAA diameter, suggesting that miR-28-5p could serve as a valuable indicator for the observation and follow-up of AAA. Additionally, highly-expressed miR-28-5p increased the risk of post-EVAR death, with postoperative miR-28-5p expression demonstrating better predictive efficacy, which has not been previously reported. In our study, the follow-up time was relatively short. However, previous studies have manifested that the prognosis of EVAR surgery is generally good [25, 26]. Also, there is various literature reporting that the post-EVAR follow-up time was 48 months or less than 48 months [27–30]. As a result, this study can provide some feedback on the correlation between miR-28-5p and post-EVAR tumor status in AAA patients, to a certain extent. Additionally, we chose 3 months as the detection time point, mainly considering the first routine follow-up at 3 months post-operation, and the follow-up period of 48 months was mainly because 120 AAA patients who attended The Quzhou Affiliated Hospital of Wenzhou Medical University, Quzhou People’s Hospital and treated with EVAR from January 2018 to December 2019 were included in this study, considering that improvements in surgical equipment, concepts, and methods of preoperative evaluation were made during that time period compared to previous ones, and the surgical team personnel in the author’s unit agreed during this period that the consistent concept of treatment plan was conducive to research. Besides, the prognosis of EVAR surgery is generally good [25, 26]. It has also been reported that EVAR is followed up for 48 months or less than 48 months after surgery [27–30]. Therefore, to a certain extent, this study can reflect the survival or death of AAA patients after EVAR. In the future research, we will extend the follow-up time to further study the survival or death of AAA patients post-EVAR.

To conclude, this study demonstrates that EVAR leads to a decrease in serum miR-28-5p expression in AAA patients. Furthermore, it reveals a positive correlation between postoperative serum levels of miR-28-5p and its preoperative and postoperative changes with AAA diameter, providing a novel indicator for evaluating AAA. However, miRNAs exhibit differential expression patterns in serum [31], plasma, and anaerobic tissues of patients with AAA [32, 33]. The process of obtaining tissue samples poses challenges and causes significant distress for patients. Conversely, peripheral blood detection is minimally invasive, easy to obtain, and can be dynamically monitored. Our study offers the possibility of identifying more convenient and sensitive markers for AAA patients in post-EVAR clinical practice.

This study only investigated pre- and post-serum miR-28-5p levels and did not compare its expression levels in different bodily fluids or tissues. Moreover, there were a high proportion of females (32.5%) due to the limited number of AAA patients included, which might limit the generalizability of the results herein. For this investigation, we used telephone communication to ascertain the mortality outcomes of patients, without delving into the associated complications. In future investigations, we will persist in enhancing our research. miRNAs exhibit differential expression in aortic tissues and blood samples obtained from AAA patients, as compared to the control group [13, 18]. According to previous report, miR-28-5p is highly expressed in AAA [14]. miRNAs can be detected in tissues or circulating blood and are highly stable during storage, making them potential ideal diagnostic or prognostic markers for AAA [18]. miR-183 and miR-141 can be used as predictors of prognosis in patients with infectious AAA [34]. In this study, the analysis found that the AUC of postoperative serum miR-28-5p for predicting postoperative death was 0.900, the sensitivity was 79.82%, and the specificity was 90.91%. Due to the limited number of cases collected in this experiment and the short follow-up time, the prediction results are still limited. Furthermore, we measured the AAA diameters on the day of EVAR operation and 3 months after surgery in patients with AAA, which was consistent with the time of blood sample collection. Diameter seems to have changed a lot. The prognosis of AAA patients after EVAR surgery is generally good [25, 26]. The thrombus formed between the pouches will be slowly absorbed, and the AAA diameter will shrink or its progress will slow down. In the case of EVAR, AAA growth is regarded as indicative of therapeutic failure since it indicates increased stress on the aneurysm wall; however, despite the intervention, the aneurysm continues to be susceptible to rupture [35]. Stable or reduced aneurysm diameter post-EVAR is traditionally deemed a successful treatment [36]. In this study, the number of cases collected is limited, and the results of this study are still limited. In the future, we will increase the number of cases for in-depth study. These aspects will be addressed in future research.

### Electronic supplementary material

Below is the link to the electronic supplementary material.

Supplementary Material 1

## Data Availability

All data generated or analysed during this study are included in this article. Further enquiries can be directed to the corresponding author.
