# Supplementary material for: Postoperative serum mir-28-5p level has predictive value for the prognosis after endovascular abdominal aortic aneurysm repair
Source: J Cardiothorac Surg. 2024 Apr 25;19:267. doi: 10.1186/s13019-024-02758-z (PMC11044549; doi:10.1186/s13019-024-02758-z)
Supplement: Supplementary file 1 — Supplementary Material 1 [file 13019_2024_2758_MOESM1_ESM.docx]

**Supplementary Table 1** Clinical baseline characteristics of the enrolled population

| Parameters | Control | AAA | *P*a |
| --- | --- | --- | --- |
|  | (n = 100) | (n = 120) |  |
| Sex (male/female) | 65/35 | 81/39 | 0.6959 |
| Age | 68.07 ± 7.28 | 68.85 ± 8.75 | 0.5365 |
| BMI (kg/m^2^) | 22.95 ± 2.24 | 23.10 ± 2.00 | 0.6009 |
| Smoking history, cases (%) | 42 (42.00%) | 55 (45.83%) | 0.5685 |
| History of Drinking Alcohol, cases (%) | 50 (50.00%) | 65 (54.17%) | 0.5378 |
| Hyperlipidemia, cases (%) | 14 (14.00%) | 20 (16.67%) | 0.5859 |
| Diabetes, cases (%) | 25 (25.00%) | 32 (26.67%) | 0.7788 |
| Chronic obstructive pulmonary disease, cases (%) | 20 (19.00%) | 24 (20.00%) | 0.8523 |
| Hypertension, cases (%) | 33 (33.00%) | 41 (34.17%) | 0.8553 |
| Coronary heart disease, cases (%) | 21 (19.00%) | 26 (21.67%) | 0.6254 |
| Framingham risk score | 21.00 (17.00, 24.00) | 21.00 (16.00, 24.00) | 0.1996 |

Note: The data were expressed as numbers or percentages, and the Chi-square test was used for comparisons between groups. Data were presented as mean ± standard deviation, and the comparisons between the two groups were performed by *t* test. Measurement data of non-normal distribution were expressed as the median (minimum, maximum), and the Mann-Whitney U test was used between the two groups.
